# Genome-Wide Association Study Reveals Novel Genomic Regions Associated With High Grain Protein Content in Wheat Lines Derived From Wild Emmer Wheat

**DOI:** 10.3389/fpls.2019.00464

**Published:** 2019-04-16

**Authors:** Jia Liu, Lin Huang, Changquan Wang, Yaxi Liu, Zehong Yan, Zhenzhen Wang, Lan Xiang, Xiaoying Zhong, Fangyi Gong, Youliang Zheng, Dengcai Liu, Bihua Wu

**Affiliations:** ^1^Triticeae Research Institute, Sichuan Agricultural University, Chengdu, China; ^2^Key Laboratory of Crop Genetic Resources and Improvement, Ministry of Education, Sichuan Agricultural University, Chengdu, China; ^3^College of Resources, Sichuan Agricultural University, Chengdu, China

**Keywords:** wild emmer wheat, common wheat, GPC, wide hybridization, GWAS

## Abstract

Grain protein content (GPC) and yield are of two important traits in wheat, but their negative correlation has hampered their simultaneous improvement in conventional breeding. Wild emmer wheat (*Triticum turgidum* ssp. *dicoccoides*) is an important genetic resource for wheat quality improvement. In this study, we report a genome-wide association study (GWAS) using 13116 DArT-seq markers to characterize GPC in 161 wheat lines derived from wild emmer. Using a general linear model, we identified 141 markers that were significantly associated with GPC, and grouped into 48 QTL regions. Using both general linear model and mixed linear model, we identified four significant markers that were grouped into two novel QTL regions on chromosomes 2BS (*QGpc.cd1-2B.1*) and 7BL (*QGpc.cd1-7B.2*). The two QTLs have no negative effects on thousand kernel weight (TKW) and should be useful for simultaneous improvement of GPC and TKW in wheat breeding. Searches of public databases revealed 61 putative candidate/flanking genes related to GPC. The putative proteins of interest were grouped in four main categories: enzymes, kinase proteins, metal transport-related proteins, and disease resistance proteins. The linked markers and associated candidate genes provide essential information for cloning genes related to high GPC and performing marker-assisted breeding in wheat.

## Introduction

Wheat provides approximately 20% of calories and 25% of proteins in the human diet. The nutritional quality of wheat grains has a significant impact on human health and well-being. The grain protein content (GPC) is an important trait for both nutritional value and end-use quality of wheat (Veraverbeke and Delcour, 2002). While the protein and micronutrients levels in modern wheat grains are inherently low and therefore, breeding for improvements in the nutritional quality of wheat, such as increased protein and micronutrient levels in the grain are possible and have the potential to alleviate hunger and nutrient deficiencies.

Breeding wheat varieties with high in both GPC and grain yield is desirable (Zhao et al., 2009). However, the GPC was negative correlated with grain yield, where phenotypic correlations for these two traits usually range between −0.30 and −0.60 (Klindworth et al., 2009). This characteristic has hampered the simultaneous improvement of these two traits in the conventional wheat breeding. The transfer of alien genes from wheat related species has become an effective approach in the development of new wheat varieties for high GPC or yield.

Wild emmer wheat (*Triticum turgidum* ssp. *dicoccoides*, 2n = 4x = AABB) is the tetraploid progenitor of cultivated wheat, offering wide genotypic variations relevant for improvement of various agronomic traits in wheat (Cakmak et al., 2004; Peleg et al., 2008b; Gomez-Becerra et al., 2010), such as GPC (e.g., Uauy et al., 2006a; Wang et al., 2018), disease resistance (Hua et al., 2009; Li et al., 2009), and drought resistance (Peleg et al., 2005, 2008a). The introgression is feasible due to the occurrence of homologous recombination between the A and B genomes of wild emmer and modern wheat.

Dissection of the genetic control of GPC in wild emmer wheat was conducted using tetraploid wheat mapping population, derived from a cross between durum wheat (cv. Langdon) and wild emmer accessions (G18-16 or FA-15-3) (Joppa et al., 1997; Gonzalez-Hernandez et al., 2004; Peleg et al., 2009). Although several quantitative trait loci (QTLs) affecting GPC were reported from these two accessions, the introgression and characterization of wild emmer GPC-QTL in a hexaploid wheat background have been less reported. The most important wild emmer GPC-QTL is the *Gpc-B1* on chromosome 6BS. This gene encodes a NAC-domain transcription factor that accelerates senescence and increases nutrient remobilization from leaves to developing grains (Uauy et al., 2006b). The introgression of *Gpc-B1* in breeding programs has shown significant improvement in GPC (Kumar et al., 2011; Mishra et al., 2015; Vishwakarma et al., 2016). However, the presence of *Gpc-B1* was associated with reductions in grain weight and yield in some wheat varieties and environments (Uauy et al., 2006a; Brevis and Dubcovsky, 2010; Tabbita et al., 2013). Therefore, the exploring of QTLs for GPC with less negative effect on yield-related traits is required.

In recent years, genome-wide association analysis (GWAS) based on linkage disequilibrium (LD) has been extensively used to decipher the genetic bases of complex traits in crops (Su et al., 2016; Wang et al., 2016; Ates et al., 2018). Advantages of GWAS over traditional QTL mapping include high resolution mapping, cost and time efficiency, and the potential to utilize large sets of germplasm resources such as landraces, elite cultivars, and advanced breeding lines. Besides, GWAS was available to analysis traits in multi-parent populations consisted of backbone parent and its derived lines in Yu and Tian (2012); Yu et al. (2014); Xiao et al. (2016). In wheat, this approach has been widely used to identify loci controlling disease resistance (Joukhadar et al., 2013; Kollers et al., 2014; Liu et al., 2017a), yield-related traits (Breseghello and Sorrells, 2006; Tadesse et al., 2015), end-use quality traits (Battenfield et al., 2016), root traits (Beyer et al., 2018), and grain zinc concentration (Velu et al., 2018).

In our previous work, the agronomically stable advanced wheat lines were obtained from common wheat cultivar Chuannong 16 (CN16 hereafter) as female crossed with wild emmer accession D1 as male through successive selfing. We found that majority of the tested wheat lines showed higher chlorophyll contents and lower chlorophyll degradation rates in flag leaves than the controls (Liu et al., 2016). Several wheat lines without *Gpc-B1* gene showed thousand-kernel weight (TKW) and GPC simultaneous improvement (Wang, 2015), implying the presence of novel loci conferring high GPC and TKW.

In the current study, GWAS was used to study genetic basis of GPC in a multi-parent population which consisted of wild emmer as backbone parent and its derived wheat lines. The objectives of this study were to characterize the wild emmer GPC loci that associated with no reductions in TKW in a hexaploid wheat population, and to scan associated candidate genes using recently published wheat reference sequences (Avni et al., 2017; Appels et al., 2018). The identified genes and markers will provide important information for cloning GPC-related genes and be useful in marker-assisted breeding for enhanced GPC in wheat.

## Materials and Methods

### Plant Materials

The low-gluten wheat cultivar CN16 (*T. aestivum*, AABBDD, 2n = 6x = 42) was crossed as the female parent with a high-protein-content wild emmer accession D1 (originating from Israel) as the male parent. The resulting pentaploid F_1_ hybrid was advanced to F_12_ to generate 106 first recombinant inbred lines (RILs) include 106 individuals with a number of stable 42 chromosomes. Some RILs with high GPC were selected and crossed to each of low/medium-gluten common wheat varieties Mianmai 46 (MM46), Chuanmai 50 (CM50), Kechengmai 2 (KCM2), and Chuanyu 18 (CY18)/YunB 58863 (YB58863) and eight generations of self-fertilization to create a second RILs with 55 individuals (Supplementary Table S1). A total of 161 wheat lines were used in this study. All of the materials were maintained at the Triticeae Research Institute, Sichuan Agricultural University, China. Wheat plants were grown in a randomized complete block design with three replicates over two growing seasons (2015 and 2016) at Chongzhou (2015CZ and 2016CZ) and one growing season (2015) at Wenjiang (2015WJ). Individual plants were spaced 10 cm apart within a 2 m row, with 30 cm between rows. Each replicate contained twenty individuals in a 2 m row. Mature seeds were harvested from the middle six plants of each row and used for GPC and TKW measurement.

### Determination of Grain Protein Content and Thousand Kernel Weight

Wheat seeds were dried to constant weight and ground to a fine powder with a Chopin CD1 AUTO (Renault, Boulogne-Billancourt, France) (Jirsa et al., 2008). A 0.5 g powder samples were measured for GPC by1241 Grain Analyzer (FOSS A/S, Hillerød, Denmark) following the method as described by Pettersson and Eckersten (2007). TKW was evaluated with an electronic balance by weighing three samples of 300 kernels (GB T 5519-2008, 2008). Analyses of variance (ANOVA) and pearson correlation coefficients for measured traits were performed using the SPSS version 22.0 (SPSS Inc., Chicago, IL, United States).

### Genotyping and Linkage Disequilibrium and Population Structure Analysis

The genomic DNA was extracted using CTAB method (Murray and Thompson, 1980). All of the tested materials were genotyped using the DArT (Diversity Arrays Technology, Canberra, ACT, Australia^1^). The obtained DArT markers data were filtered based on call rate (minimum threshold value of 85%) and reproducibility (minimum threshold value of 95%). A total of 24022 DArT markers were recalled from marker data. For the further analysis, markers with missing data greater than 10% and minor allele frequency (MAF) less than 5% were filtered, which resulted in 13116 DArT markers were retained and used. Linkage disequilibrium (LD) analysis was performed for each of the 21 wheat chromosomes associated with mapped DArT markers using software TASSEL 3.0 (Bradbury et al., 2007). The LD squared allele-frequency correlation (*r*^2^), which contains both mutational and recombination history information, was evaluated for syntenic loci (*p* < 0.001). The mean *r*^2^ over different genetic distances was calculated for the A, B, and D subgenomes as well as the whole genome. The LD decay plots were generated using *r*^2^ and the genetic map distance between markers.

Population structure was estimated using Structure software 2.3.4 (Pritchard et al., 2000). An admixture model with ten replicates for each number of genetic groups (*K* = 1–10) and 100,000 iterations of burn-in followed by 100,000 Markov Chain Monte Carlo (MCMC) iterations were used. The outputs of the genetic group analysis were extracted in Structure Harvester (Earl and von Holdt, 2012). The optimal *K*-value was determined using the delta K method as described by Evanno et al. (2005). Furthermore, principal component analysis (PCA) of filtered markers was performed with the TASSEL 3.0 (Bradbury et al., 2007) and the first two PCA values were plotted.

### Genome-Wide Association Study for GPC

To eliminate the environmental impact, the best linear unbiased prediction (BLUP) across all tested environments was carried out using the META-R (Alvarado et al., 2015). The association analysis of DArT markers and GPC was performed using a general linear model (GLM) and mixed linear model (MLM) in TASSEL. The first three PCA values were used as covariate in the model to adjust population stratification and kinship matrix (K) was calculated using Scaled IBS method (Yu et al., 2006; Liu et al., 2018). A Bonferroni-corrected *p* value threshold at α = 1 was used as the cutoff (Yang et al., 2014; Liu et al., 2017b). For the 13116 DArT markers, the *p*-value threshold at α = 1 was 7.62 × 10^−5^, with a corresponding −log_10_*p* value of 4.12. Significant markers were visualized with a Manhattan plot using Haploview 4.2 software (Barrett et al., 2004). Important *p*-value distributions (expected vs. observed *p*-values on a −log10 scale) were shown with a quantile-quantile plot. To identify the candidate genes linked to significant markers, we performed a BLAST search against the International Wheat Genome Sequencing Consortium database (IWGSC^2^) and the International Wild Emmer Wheat Genome Sequencing Consortium database^3^ using the significant marker sequences. When a DArT marker sequence and a wheat contig were 100% identical, the sequence was extended to 5 kb for each marker using the IWGSC BLAST results (Liu et al., 2017b). The extended sequence was explored to predict flanking genes using BLAST search against the Triticeae Multi-omics center database^4^.

## Results

### Variation for GPC in the Wheat Lines Derived From Wild Emmer

**FIGURE 1:**
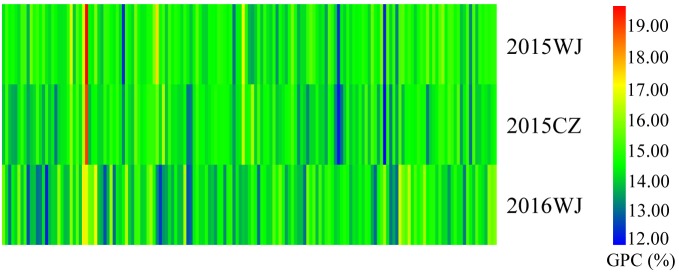
GPC analysis of the wheat lines derived from wild emmer grown at 2015WJ, 2015CZ, and 2016WJ.

**FIGURE 2:**
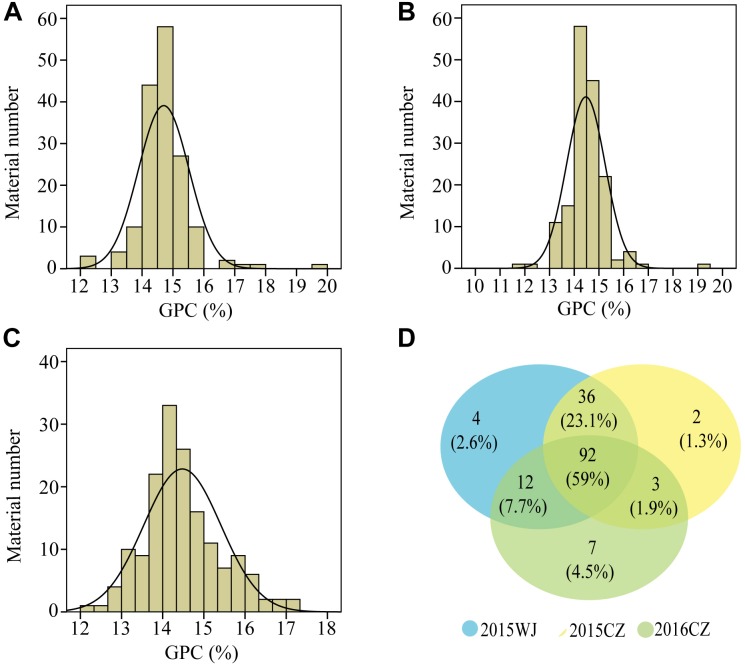
Frequency distributions of GPC values under three environments in the wheat lines derived from wild emmer. **(A)**, 2015WJ; **(B)**, 2015CZ; **(C)**, 2016CZ; **(D)** Wayne chart: the number of wheat lines with GPC ≥14% in tested environments (Blue color, 2015WJ; Yellow color, 2015CZ; Green color, 2016CZ).

The variation for GPC in the wheat lines and their core parents were summarized in Table 1. Most genotypes displayed relatively stable GPC across the three environments (2015WJ, 2015CZ, and 2016CZ) (Figure 1). The GPC was highly correlated (*p* < 0.01) across environments (*r* = 0.210–0.732) based on the two-tailed pearson product-moment correlation coefficient test (Fieller et al., 1957; Table 2). The male parent D1 exhibited significantly higher GPC (mean range 22.45–23.43%) compared to female parent CN16 (mean range 12.00–12.90%) across all test environments (Table 1). The GPC of the wheat lines derived from wild emmer were ranged from 11.97 to 19.73% (mean range 14.47–14.70%) across three environments. The lowest mean GPC (11.97%) was recorded in 2015 at Chongzhou, whereas the highest mean GPC (19.73%) was recorded in 2015 at Wenjiang. Most of the wheat lines showed significantly higher GPC than CN16 (Table 1 and Figure 2). A large number of wheat lines had GPC ranged from 14 to 15% in each environment (Figure 2A–C). A total of 92 (59%) wheat lines displayed consistently high GPC (≥14%) across three tested environments (Figure 2D). The mean TKW of wheat lines were higher than that of female parent CN16 under 2015WJ, 2015CZ, and 2016CZ environments (Table 1). Pearson’s correlation analysis showed that there was no significant negative correlation between GPC and TKW across environments (Table 2). Therefore, according to the phenotypic BLUP data of GPC and TKW, a total of 132 wheat lines with strong gluten (GPC ≥14%) and high TKW (≥46.24 g) were identified (Supplementary Figure S1).

**Table 1:** The variation for GPC and TKW in wheat lines derived from wild emmer under three environments.

Trait	Environment	Parents		Derived lines		
		CN16	D1	Mean ± SD	Range	CV (%)
GPC	2015WJ	12.89^a^ ± 0.25	22.45^c^ ± 0.44	14.70^b^ ± 0.82	12.35–19.73	5.58
	2015CZ	12.00^a^ ± 0.10	22.96^c^ ± 0.46	14.47^b^ ± 0.78	11.97–19.18	5.39
	2016CZ	12.90^a^ ± 0.08	23.43^c^ ± 0.94	14.48^b^ ± 0.94	12.25–17.33	6.49
TKW	2015WJ	43.19^b^ ± 4.99	19.21^a^ ± 4.39	45.78^b^ ± 4.94	31.35–57.00	10.79
	2015CZ	49.33^b^ ± 1.54	15.00^a^ ± 0.67	52.82^b^ ± 4.40	37.08–64.63	8.33
	2016CZ	42.70^b^ ± 4.97	31.63^a^ ± 0.71	48.94^c^ ± 4.55	35.00–60.88	9.30

*The small letters a, b, and c indicate the significant differences at the 0.05 level. CV, coefficient of variation.*

**Table 2:** Pearson correlation coefficient of GPC and TKW among all measured environments in the wheat lines derived from wild emmer.

Correlations	2015WJ GPC	2015CZ GPC	2016CZ GPC	2015WJ TKW	2015CZ TKW
2015CZ GPC	0.732^∗∗^				
2016CZ GPC	0.263^∗∗^	0.210^∗∗^			
2015WJ TKW	−0.02	−0.035	0.171^∗^		
2015CZ TKW	−0.017	0.115	0.129	0.436^∗∗^	
2016CZ TKW	0.029	0.135	−0.062	0.031	0.367^∗∗^

*^∗^Correlation is significant at the 0.05 level (2-tailed). ^∗∗^Correlation is significant at the 0.01 level (2-tailed).*

### Analysis of DArT Markers, Population Structure and Linkage Disequilibrium (LD)

A total of 13116 polymorphic DArT markers (MAF≥5% and missing ≤10%) were used to estimate the underlying population structure. Of these, 4710, 6718, and 1688 markers were mapped on the A, B, and D genomes, respectively. The total map length was 5941.15 cM, and the average distance between markers for the A, B, and D genomes was 0.44 cM, 0.30 cM, and 1.11 cM, respectively (Supplementary Table S2). Chromosome 1B contained the largest number of markers (1708) and the average distance between markers was 0.33 cM, whereas Chromosome 4D contained the smallest number of markers (52) and the average distance between markers was 3.47 cM.

Base on structure analysis, the highest delta *K*-value was 3, the 163 genotypes were divided into three main sub-groups, namely Gp1, Gp2, and Gp3, representing 41, 75, and 47 genotypes, respectively. Gp1 only included individuals from the 106 RILs; Gp2 included individuals from both RIL populations; the two core parents and most of the individuals from the 55 RILs were belonged to Gp3 (Supplementary Figure S2A and Supplementary Table S1). PCA was also performed to investigate population structure. The first two PCs explained 12.64 and 7.54% variation in the population of wheat lines (Supplementary Figure S2B). The PCA results were basically agreed with that of structure analysis, which clearly divided the genotypes into three main sub-groups.

LD between pairs of markers was estimated for each of the 21 chromosomes (Supplementary Table S3). In the whole genome, 55.55% of pairwise DArT markers had a significant LD (*p* < 0.001), and 37.37% of significant pairwise markers had an *r*^2^ > 0.2. In the A, B, and D subgenomes, 51.12, 62.58, and 39.71% of pairwise DArT markers had a significant LD (*p* < 0.001), and 32.06, 43.90, and 25.98% of the significant pairwise markers had an *r*^2^ > 0.2, respectively. The extent of LD in each chromosome was different. Chromosome 1B showed the highest percentage of pairwise markers with significant LDs (75.64%), with *r*^2^ > 0.2 (62.14% of significant pairwise markers). In contrast, chromosome 4D had the lowest percentage of pairwise markers with significant LDs (17.58%), with *r*^2^ > 0.2 (7.35% of significant pairwise markers). The mean *r*^2^-values for the A, B, and D genomes as well as the whole genome were rapidly decreased with increasing pairwise distance (Supplementary Figure S3). The LD decay distances were approximately 9, 12, 13, and 12 cM for the A, B, and D genomes as well as the whole genome, respectively (Supplementary Figure S3).

### GWAS for GPC in the Wheat Lines Derived From Wild Emmer

**FIGURE 3:**
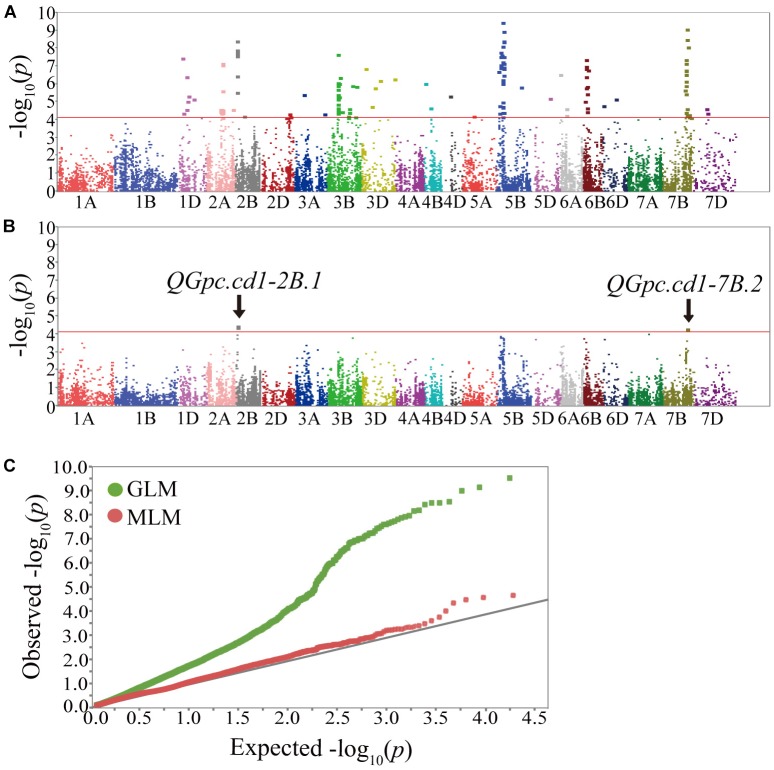
Manhattan plots of genome-wide association scan for GPC in three environments. Manhattan plot showing –log_10_ *p* values of the markers for GPC. Red lines indicate the −log_10_ *p* threshold of 4.12. Chromosomes carrying significant markers detected by GLM **(A)** and MLM **(B)**; **(C)**, Quantile-quantile plots of *GPC*. Green plot, GLM model; Red plot, MLM model; Black line, the expected values.

A total of 141 markers had significant results in the three environments by GLM with PVEs ranged from 9.90 to 22.79% (Supplementary Table S4). These markers were distributed on all wheat chromosomes except chromosomes 1A, 1B, 4A, and 7A (Figure 3A). In the MLM, four significant markers (three on 2B and one on 7B) were detected with PVEs of 11.92–12.93%, and these markers were also detected by GLM (Figure 3B and Tables 3, 4). To determine the confidence interval for the potential QTL identified in this study, markers significantly associated to the GPC that were co-locating together and/or adjacent within intervals inferior to the LD decay distance of ± 5 cM from the association peak were considered as a same QTL region. In total, the 141 significant markers detected were grouped into 48 loci (Supplementary Table S4). Of these, four significant markers by both models were grouped into two loci on chromosomes 2B (*QGpc.cd1-2B.1*) and 7B (*QGpc.cd1-7B.2*) (Table 3 and Figure 3B) that potentially contain GPC genes. In particular, the mean PVE of three markers on chromosome 2B in these two stable major loci was 12.78 and 20.49% in MLM and GLM, and the PVE value of marker 1166404 located on chromosome 7B was the highest at 22.79% in GLM. Quantile-quantile plots of *p*-values comparing the uniform distribution of the expected −log_10_*p* value to the observed −log_10_*p* value for GPC showed that the MLM was more conservative than GLM (Figure 3C).

**Table 3:** Significant DArT markers for GPC identified by MLM and GLM.

QTL	Marker	Chr.	Position (cM)	Environment	MLM		GLM	
					−log10(*p*)	PVE (%)	−log10(*p*)	PVE (%)
*QGpc.cd1-2B.1*	1116535	2B	3.93	BLUP	4.39	12.55	7.84	20.19
	3022213	2B	8.70	BLUP	4.47	12.93	8.42	21.35
	1088696	2B	8.70	BLUP	4.44	12.87	7.89	19.93
*QGpc.cd1-7B.2*	1166404	7B	216.46	BLUP	4.29	11.92	9.07	22.79

*Chr., chromosome; PVE, phenotypic variation explained.*

**Table 4:** MTAs identified by GWAS base on the BLUP data.

Model	No. sig.^a^	Average -log(*p*)	Range -log(*p*)	Average PVE (%)	Range PVE (%)	No. Shared^b^
Mixed linear model (MLM)	4	4.4	4.29–4.47	12.57	11.92–12.93	4
General linear model (GLM)	141	5.76	4.12–9.45	14.15	9.90–22.79	

*^a^The total number of MTAs identified by GLM or MLM at the threshold of -log_10_(p) = 4.12, respectively. ^b^The number of shared significant markers. PVE, phenotypic variation explained.*

### Candidate Genes That May Be Associated With GPC

Sequences of DArT markers related with GPC were utilized to predicate candidate genes using the recently annotated wheat reference sequence (RefSeq v.1.0) and wild emmer wheat reference sequence (WEWSeq v.1.0). The enzymes and proteins related to 61 putative candidate genes were predicated (Supplementary Table S5). These candidate genes could be roughly divided into four groups according to the types of protein they encoded. The first group of candidate genes included enzymes associated with nitrogen transport, such as aspartic peptidase, peroxidase, and peptidase. The second group consisted of genes including kinase proteins. The third group included metal transport related proteins, such as zinc transporter, and heavy metal transport proteins. The last group consisted of disease resistance proteins, such as MYB transcription factor and NAC domain-containing proteins (Supplementary Table S5).

## Discussion

Wild emmer wheat has been found to show higher concentration of protein in grain when compared to common cultivated wheat (Cakmak et al., 2004). Several QTL analyses were employed to dissect the genetic basis of grain protein using a tetraploid wheat RIL population (Joppa et al., 1997; Gonzalez-Hernandez et al., 2004; Peleg et al., 2009). In this study, the 161 RILs derived from wild emmer were phenotyped in multiple environments and genotyped using the DArT markers to dissect the genetic basis of grain protein accumulation in hexaploid wheat. GWAS identified 141 significant markers for GPC by GLM, and 61 associated candidate genes were predicated using the sequences annotation (RefSeq v.1.0; WEWSeq v.1.0). Two large effect QTL regions on chromosomes 2B and 7B were detected by both GLM and MLM. Our findings are consistent with previous studies that the group 2 and 7 chromosomes harboring genes for nutritional quality traits in wheat (Crespo-Herrera et al., 2017; Velu et al., 2018).

Peleg et al. (2009) identified ten GPC-QTLs related to GPC using a mapping population derived from LDN crossed with wild emmer G18-16 that showed high GPC phenotype; these loci were distributed on 10 wild emmer chromosomes except 1A, 1B, 3A, and 4B; A few genomic regions (2A, 5A, 6B, and 7A) were found to contain clusters of QTLs for GPC. In the current study, 48 significant GPC-QTLs were scattered across all wheat chromosomes (except 1A, 1B, 4A, and 7A) using a GLM model. We found the GPC-QTLs on chromosomes 2A, 3B, and 6A are located in the same region as compared to Peleg et al. (2009) and the remaining QTLs in our studies were novel. However, GPC-QTLs on chromosomes 4A and 7A in the G18-16 tetraploid mapping population were not detected in our hexaploid RILs.

Candidate genes prediction revealed that some QTL were related to nitrogen metabolism and disease resistance. For example, QTLs on chromosome 5B were associated with glutathione S-transferase (GST) and proline transporter. Previous research showed that GSTs are critical for nitrogen fixation in plant (Dalton et al., 2009). In particular, the marker 1064158 (55.28 cM) located on chromosome 5B was consistent with the results reported by previous study (Kumar et al., 2018b). *QGpc.cd1-2A.2* and *QGpc.cd1-5A* were associated with methyltransferase, cytochrome P450 and NAC domain-containing protein. *QGpc.cd1-6A.1* were associated with nitrogenase-stabilizing. Kim et al. (2005) reported that the function of nitrogenase (NA) is closely related to plant nitrogen metabolism, and nitrogenase activity has a positive effect on wheat growth, total plant N-yield, and protein content. Moreover, although *QGpc.cd1-1D.1* was only detected under one model, it showed a very high PVE value 19.06% and was associated with disease resistance protein. Note worthily, we detected two QTLs *QGpc.cd1-6B.2* and *QGpc.cd1-6B.3* on 6BS that associated with zinc transporter and glutenin macropolymer (GMP) synthase. In barley, the zinc transporter (for example *HvMTP1*) was associated with enhancement of grain Zn content (Menguer et al., 2018). The glutenin macropolymer (GMP) content of flour was confirmed to be significant related to high protein content (Weegels et al., 1996). Uauy et al. (2006b) reported the *Gpc-B1* on 6BS that was associated with N, Zn, and Fe remobilization in plants. However, this locus was not detected in the current study and the physical position of the two QTLs on 6BS was larger than 10 Mb away from the functional *Gpc-B1.* Different hypotheses may explain this observation: (1) the relatively low density of DArT markers; (2) the low frequency of *Gpc-B1* in the current wheat lines that cannot detected by GWAS. Additional studies are required to test these hypotheses.

Using a MLM model, we identified two significant stable GPC-QTLs *QGpc.cd1-2B.1* (3.93–8.70 cM) and *QGpc.cd1-7B.2* (216.46 cM), explaining 11.92–12.93 and 19.93–22.79% of the phenotypic variance. A GPC-QTL (95.4 ± 13.8 cM) on wild emmer chromosome 2B was detected by Peleg et al. (2009). In durum wheat, Suprayogi et al. (2009) identified a QTL on 2B which was associated with higher GPC in all tested environments. Recently, two environmental stable GPC-QTLs *QGpc.mna-2B* (Tsilo et al., 2010) and *QGpc.2B-yume* (Terasawa et al., 2016) were reported in wheat. *QGpc.mna-2B* was located between the markers *Xwmc245-Xgwm271* at position 64.0–65.0 cM on chromosome 2B; whereas *QGpc.2B-yume* was sited near the marker *Xgpw4382* at position 67.1 cM. The position of *QGpc.cd1-2B.1* (3.93–8.70 cM) in our study is different to the previous reported GPC-QTLs on chromosome 2B. On chromosome 7B, at least five GPC-QTLs with stable effects across environments have been reported in different studies (reviewed by Kumar et al., 2018a). Of these, most GPC-QTLs were located on the short arm of chromosome 7B. Blanco et al. (2006) reported a QTL located on 7BL explained 9.1% of the phenotypic variance of the GPC which close to the SSR marker *Xgwm577* at position 137 cM. *QGpc.cd1-7B.2* in our study is located at position 216.46 cM on the long arm of chromosome 7B. Therefore, we concluded that the two wild emmer QTLs on 2B and 7B conferring high GPC were novel.

In this study, the highly associated markers sequences were used to predicate putative candidate genes for *QGpc.cd1-2B.1* and *QGpc.cd1-7B.2.* We found that the ABA-responsive binding factor and processing peptidase genes may play crucial roles in conferring of higher protein content in wheat grain. For example, the ABA-dependent NAC transcription factor (*Os*NAC5) in rice was reported to be associated with Fe, Zn and amino acids remobilization from green tissues to seeds (Sperotto et al., 2009). In addition, the *QGpc.cd1-7B.2* was associated with processing peptidase. Peptidases are key enzymes that involved in nitrogen metabolism, such as nitrate reductase, endopeptidase, aminopeptidase, and carboxypeptidase. It was known that leaf senescence and nitrogen metabolism of senescing tissues are two important factors that determining the GPC in cereals (Jukanti and Fischer, 2008). Moreover, the peptidase plays a role in regulating the redistribution and utilization of nitrogen in wheat plants that affects the grain protein quality of wheat (Waters et al., 1980). Functional analyses are needed to further understand the pathways of these candidate genes for regulating high GPC in wheat.

The wheat grain yield and GPC are negatively correlated and the negative correlation was mainly due to the dilution effect that making the simultaneous increase of the two traits challenging (Cox et al., 1985; Day et al., 1985; Simmonds, 1995). In our previous studies, we confirmed that the introgression of high GPC and TKW traits from wild emmer wheat to common wheat is feasible by wide hybridization (Wu et al., 2008; Wang et al., 2018). The TKW was positively correlated with grain yield as reported by previous studies (Bhatta et al., 2018a,b). In the present study, no significant negative correlation between GPC and TKW were detected in the wheat lines derived from wild emmer, suggesting presence of novel GPC-QTLs that are not associated or less associated with TKW. Our results indicate the improvement of GPC without sacrificing yield could be possible by dissection and introgression of wild emmer QTLs. In addition, our study indicates the potential value of *QGpc.cd1-2B.1* and *QGpc.cd1-7B.2* for GPC improvement and provides comprehensive use of wild emmer wheat for offering new source of alleles to increase GPC in wheat.

## Conclusion

Wild emmer is a valuable resource for wheat quality improvement. The wild emmer derived wheat population showed high GPC and a weak correlation between GPC and TKW, indicating simultaneous improvement of GPC and TKW could be possible. A GWAS identified 48 QTLs (141 MTAs), of which two novel major QTL regions (chromosomes 2B and 7B) for high GPC were stable detected in two models. The QTLs have no negative effects on TKW and would facilitate the development of cultivars with both high GPC and TKW. Furthermore, at significant loci and flanking regions, we identified 61 putative candidate genes that might play crucial roles in conferring of higher protein content in wheat grain. The identified markers and genes offer information for cloning genes related to GPC and may be used in wheat breeding programs.

## Author Contributions

BW, JL, and LH designed the study. JL, ZW, LX, XZ, and FG carried out experiments. JL analyzed the data and drafted the manuscript. CW, YL, and ZY provided the genotyping data. YZ and DL provided their constructive comments and suggestions. LH, BW, DL, and YZ revised the manuscript. All authors read and approved the final manuscript.

## Conflict of Interest Statement

The authors declare that the research was conducted in the absence of any commercial or financial relationships that could be construed as a potential conflict of interest.

## Footnotes

1 http://www.DiversityArrays.com/

2 http://www.wheatgenome.org/

3 http://wewseq.wixsite.com/consortium

4 http://202.194.139.32/
