# Supplementary material for: Genome-Wide Association Study Reveals Novel Genomic Regions Associated With High Grain Protein Content in Wheat Lines Derived From Wild Emmer Wheat
Source: Front Plant Sci. 2019 Apr 16;10:464. doi: 10.3389/fpls.2019.00464 (PMC6477094; doi:10.3389/fpls.2019.00464)
Supplement: Supplementary file 1 [file Data_Sheet_1.docx]

Supplementary Material

**Genome-Wide Association Study Reveals Novel Genomic Regions Associated With High Grain Protein Content in Wheat Lines Derived From Wild Emmer Wheat**

Jia Liu^1,2†^, Lin Huang^1,2†^, Changquan Wang^3^, Yaxi Liu^1,2^, Zehong Yan^1,2^, Zhenzhen Wang^1^, LanXiang^1^, Xiaoying Zhong^1^, Fangyi Gong^1^, Youliang Zheng^1,2^, Dengcai Liu^1,2^, Bihua Wu^1,2^*

^1^Triticeae Research Institute, Sichuan Agricultural University, Chengdu, China

^2^Key Laboratory of Crop Genetic Resources and Improvement, Ministry of Education, Sichuan Agricultural University, Chengdu, China

^3^College of Resources, Sichuan Agricultural University, Chengdu, China

^†^Jia Liu and Lin Huang contributed equally to this work.

*** Correspondence:** Bihua Wu: [wubihua2017@126.com](mailto:wubihua2017@126.com)

# Supplementary Figures and Tables

## Supplementary Figures


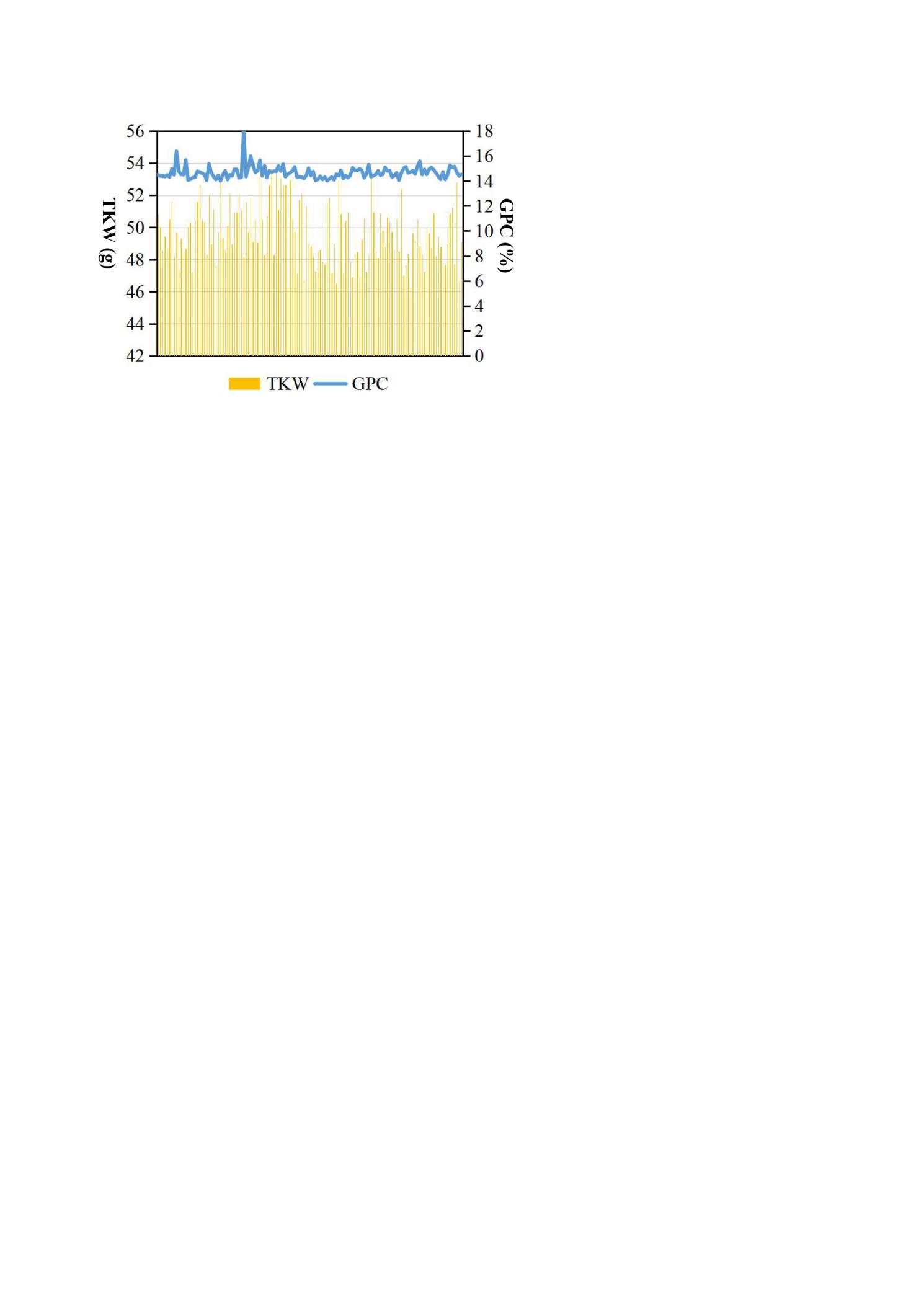


## Supplementary Figure 1.132 wheat lines with strong gluten (GPC≥14%) and high TKW (≥46.24g).


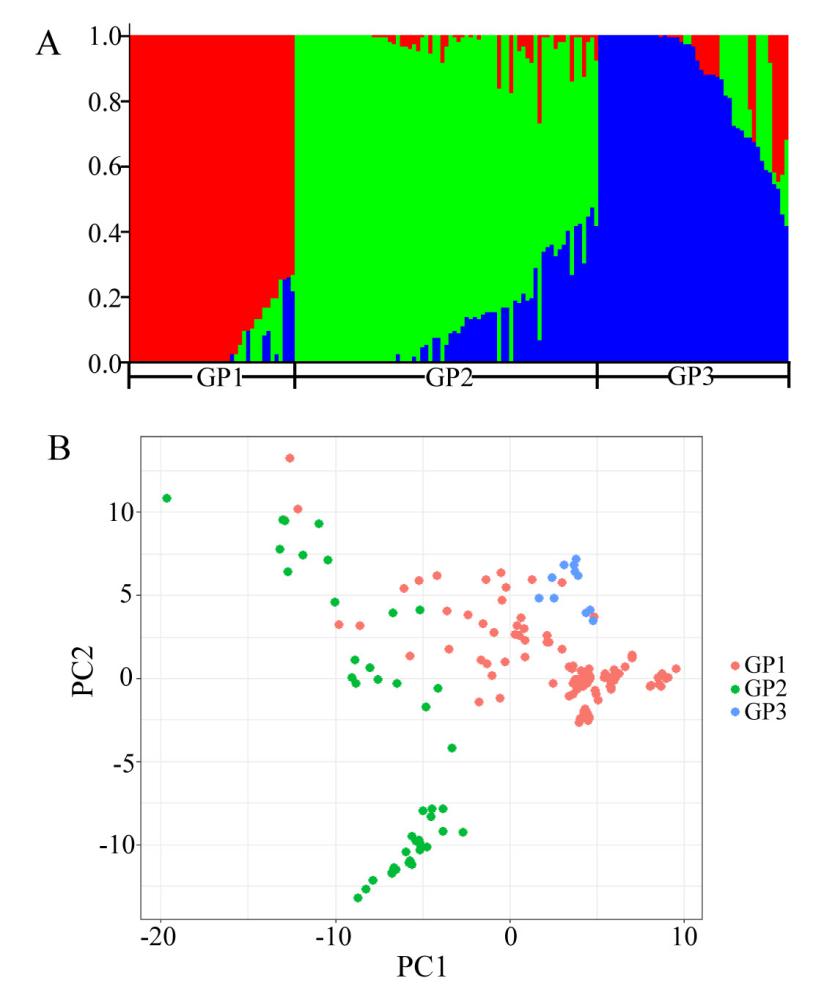


## Supplementary Figure 2.(A) Population structure of wheat lines derived from wild emmer based on DArT markers. Note: Y-axis represents the proportion of different genotype sharing the genetic background. (B) Principal component analysis of wheat lines derived from wild emmer based on DArT markers.


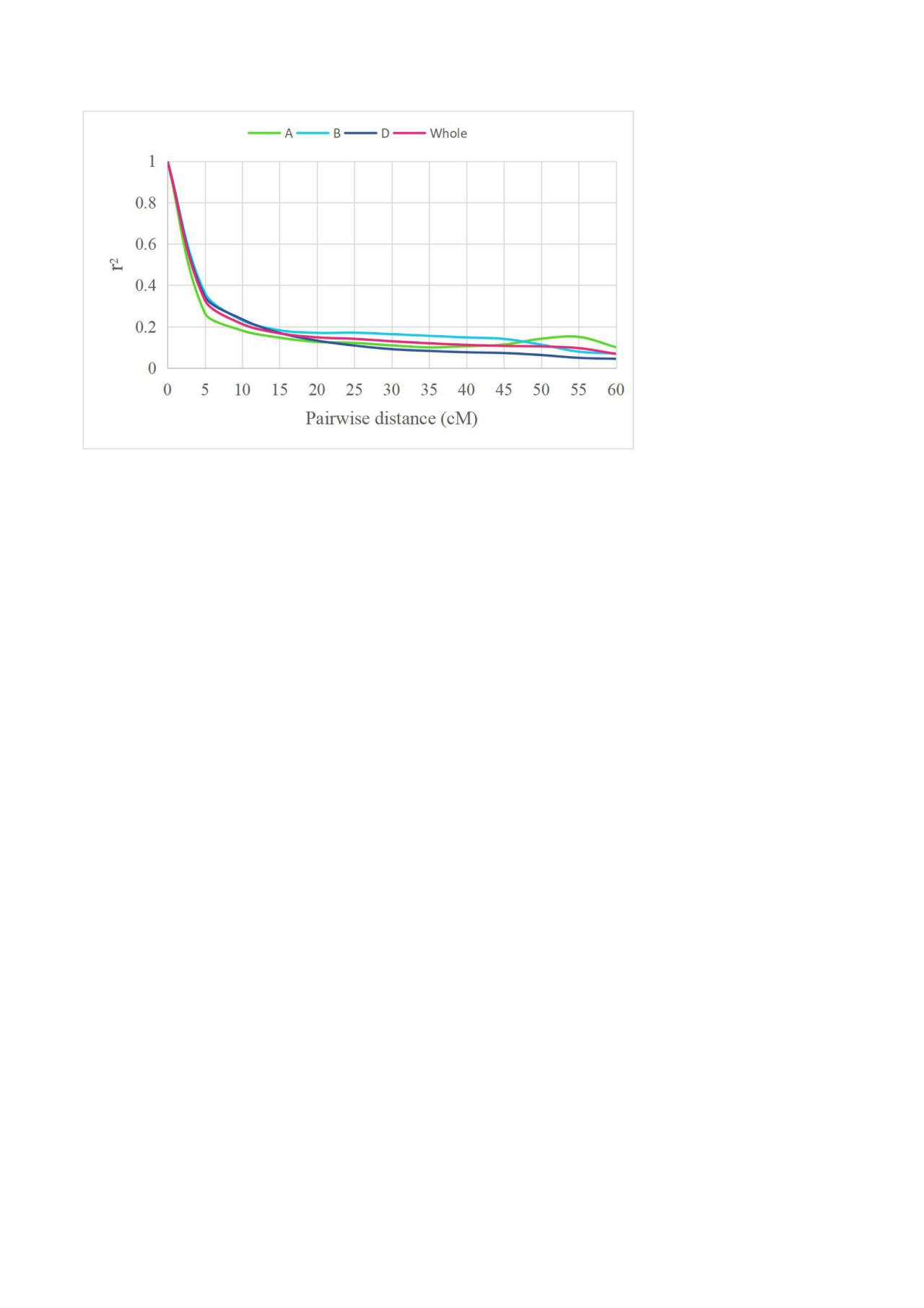


## Supplementary Figure 3.LD decay over different genetic distances (cM) for A, B and D subgenomes and whole hexaploid wheat genome in population (r^2^=0.2).

## Supplementary Tables

**Supplementary Table1.**The wheat lines used in the current study.

| Taxonomic | Accession | Pedigree | Subgroup^a^ |
| --- | --- | --- | --- |
| *Triticum aestivum* ssp. *aestivum* (AABBDD) | CN16 | Female Parent | Gp3 |
| *Triticum turgidum ssp. dicoccoides* (AABB) | D1 | Male Parent | Gp3 |
| *Triticum aestivum* ssp. *aestivum* (AABBDD) | BAd63-4 | CN16×D1 | Gp2 |
| *Triticum aestivum* ssp. *aestivum* (AABBDD) | BAd64-5 | CN16×D1 | Gp2 |
| *Triticum aestivum* ssp. *aestivum* (AABBDD) | BAd67-2 | CN16×D1 | Gp2 |
| *Triticum aestivum* ssp. *aestivum* (AABBDD) | BAd68-2 | CN16×D1 | Gp2 |
| *Triticum aestivum* ssp. *aestivum* (AABBDD) | BAd69-2 | CN16×D1 | Gp2 |
| *Triticum aestivum* ssp. *aestivum* (AABBDD) | BAd70-2 | CN16×D1 | Gp2 |
| *Triticum aestivum* ssp. *aestivum* (AABBDD) | BAd70-4 | CN16×D1 | Gp2 |
| *Triticum aestivum* ssp. *aestivum* (AABBDD) | BAd71-2 | CN16×D1 | Gp2 |
| *Triticum aestivum* ssp. *aestivum* (AABBDD) | BAd71-3 | CN16×D1 | Gp2 |
| *Triticum aestivum* ssp. *aestivum* (AABBDD) | BAd73-2 | CN16×D1 | Gp2 |
| *Triticum aestivum* ssp. *aestivum* (AABBDD) | BAd73-7 | CN16×D1 | Gp2 |
| *Triticum aestivum* ssp. *aestivum* (AABBDD) | BAd74-2 | CN16×D1 | Gp2 |
| *Triticum aestivum* ssp. *aestivum* (AABBDD) | BAd76-5 | CN16×D1 | Gp2 |
| *Triticum aestivum* ssp. *aestivum* (AABBDD) | BAd79-5 | CN16×D1 | Gp2 |
| *Triticum aestivum* ssp. *aestivum* (AABBDD) | BAd84-5 | CN16×D1 | Gp2 |
| *Triticum aestivum* ssp. *aestivum* (AABBDD) | BAd89-1 | CN16×D1 | Gp2 |
| *Triticum aestivum* ssp. *aestivum* (AABBDD) | BAd91-4 | CN16×D1 | Gp2 |
| *Triticum aestivum* ssp. *aestivum* (AABBDD) | BAd93-5 | CN16×D1 | Gp2 |
| *Triticum aestivum* ssp. *aestivum* (AABBDD) | BAd95-2 | CN16×D1 | Gp2 |
| *Triticum aestivum* ssp. *aestivum* (AABBDD) | BAd95-4 | CN16×D1 | Gp2 |
| *Triticum aestivum* ssp. *aestivum* (AABBDD) | BAd97-2 | CN16×D1 | Gp2 |
| *Triticum aestivum* ssp. *aestivum* (AABBDD) | BAd99-5 | CN16×D1 | Gp2 |
| *Triticum aestivum* ssp. *aestivum* (AABBDD) | BAd100-3 | CN16×D1 | Gp2 |
| *Triticum aestivum* ssp. *aestivum* (AABBDD) | BAd104-2 | CN16×D1 | Gp2 |
| *Triticum aestivum* ssp. *aestivum* (AABBDD) | BAd106-1 | CN16×D1 | Gp2 |
| *Triticum aestivum* ssp. *aestivum* (AABBDD) | BAd106-4 | CN16×D1 | Gp2 |
| *Triticum aestivum* ssp. *aestivum* (AABBDD) | BAd107-1 | CN16×D1 | Gp2 |
| *Triticum aestivum* ssp. *aestivum* (AABBDD) | BAd107-4 | CN16×D1 | Gp2 |
| *Triticum aestivum* ssp. *aestivum* (AABBDD) | BAd108-2 | CN16×D1 | Gp3 |
| *Triticum aestivum* ssp. *aestivum* (AABBDD) | BAd109-2 | CN16×D1 | Gp2 |
| *Triticum aestivum* ssp. *aestivum* (AABBDD) | BAd111-1 | CN16×D1 | Gp2 |
| *Triticum aestivum* ssp. *aestivum* (AABBDD) | BAd112-6 | CN16×D1 | Gp2 |
| *Triticum aestivum* ssp. *aestivum* (AABBDD) | BAd117-3 | CN16×D1 | Gp2 |
| *Triticum aestivum* ssp. *aestivum* (AABBDD) | BAd119-3 | CN16×D1 | Gp2 |
| *Triticum aestivum* ssp. *aestivum* (AABBDD) | BAd119-4 | CN16×D1 | Gp2 |
| *Triticum aestivum* ssp. *aestivum* (AABBDD) | BAd122-3 | CN16×D1 | Gp2 |
| *Triticum aestivum* ssp. *aestivum* (AABBDD) | BAd122-5 | CN16×D1 | Gp1 |
| *Triticum aestivum* ssp. *aestivum* (AABBDD) | BAd123-2 | CN16×D1 | Gp2 |
| *Triticum aestivum* ssp. *aestivum* (AABBDD) | BAd123-3 | CN16×D1 | Gp2 |
| *Triticum aestivum* ssp. *aestivum* (AABBDD) | BAd124-4 | CN16×D1 | Gp2 |
| *Triticum aestivum* ssp. *aestivum* (AABBDD) | BAd124-6 | CN16×D1 | Gp2 |
| *Triticum aestivum* ssp. *aestivum* (AABBDD) | BAd125-2 | CN16×D1 | Gp2 |
| *Triticum aestivum* ssp. *aestivum* (AABBDD) | BAd127-1 | CN16×D1 | Gp2 |
| *Triticum aestivum* ssp. *aestivum* (AABBDD) | BAd128-2 | CN16×D1 | Gp2 |
| *Triticum aestivum* ssp. *aestivum* (AABBDD) | BZn128-5 | CN16×D1 | Gp2 |
| *Triticum aestivum* ssp. *aestivum* (AABBDD) | BAd128-6 | CN16×D1 | Gp2 |
| *Triticum aestivum* ssp. *aestivum* (AABBDD) | BAd129-2 | CN16×D1 | Gp2 |
| *Triticum aestivum* ssp. *aestivum* (AABBDD) | BAd129-4 | CN16×D1 | Gp2 |
| *Triticum aestivum* ssp. *aestivum* (AABBDD) | BAd129-6 | CN16×D1 | Gp2 |
| *Triticum aestivum* ssp. *aestivum* (AABBDD) | BZn130-3 | CN16×D1 | Gp2 |
| *Triticum aestivum* ssp. *aestivum* (AABBDD) | BAd133-5 | CN16×D1 | Gp2 |
| *Triticum aestivum* ssp. *aestivum* (AABBDD) | BAd134-3 | CN16×D1 | Gp2 |
| *Triticum aestivum* ssp. *aestivum* (AABBDD) | BAd137-4 | CN16×D1 | Gp2 |
| *Triticum aestivum* ssp. *aestivum* (AABBDD) | BAd137-5 | CN16×D1 | Gp2 |
| *Triticum aestivum* ssp. *aestivum* (AABBDD) | BAd139-6 | CN16×D1 | Gp2 |
| *Triticum aestivum* ssp. *aestivum* (AABBDD) | BAd141-6 | CN16×D1 | Gp1 |
| *Triticum aestivum* ssp. *aestivum* (AABBDD) | BAd142-1 | CN16×D1 | Gp2 |
| *Triticum aestivum* ssp. *aestivum* (AABBDD) | BAd142-4 | CN16×D1 | Gp2 |
| *Triticum aestivum* ssp. *aestivum* (AABBDD) | BAd142-5 | CN16×D1 | Gp2 |
| *Triticum aestivum* ssp. *aestivum* (AABBDD) | BAd142-6 | CN16×D1 | Gp2 |
| *Triticum aestivum* ssp. *aestivum* (AABBDD) | BAd143-3 | CN16×D1 | Gp2 |
| *Triticum aestivum* ssp. *aestivum* (AABBDD) | BAd144-1 | CN16×D1 | Gp2 |
| *Triticum aestivum* ssp. *aestivum* (AABBDD) | BAd144-6 | CN16×D1 | Gp2 |
| *Triticum aestivum* ssp. *aestivum* (AABBDD) | BAd145-3 | CN16×D1 | Gp2 |
| *Triticum aestivum* ssp. *aestivum* (AABBDD) | BAd146-4 | CN16×D1 | Gp2 |
| *Triticum aestivum* ssp. *aestivum* (AABBDD) | BAd147-2 | CN16×D1 | Gp1 |
| *Triticum aestivum* ssp. *aestivum* (AABBDD) | BAd149-3 | CN16×D1 | Gp1 |
| *Triticum aestivum* ssp. *aestivum* (AABBDD) | BAd152-7 | CN16×D1 | Gp1 |
| *Triticum aestivum* ssp. *aestivum* (AABBDD) | BAd153-3 | CN16×D1 | Gp1 |
| *Triticum aestivum* ssp. *aestivum* (AABBDD) | BAd156-2 | CN16×D1 | Gp1 |
| *Triticum aestivum* ssp. *aestivum* (AABBDD) | BAd157-4 | CN16×D1 | Gp1 |
| *Triticum aestivum* ssp. *aestivum* (AABBDD) | BAd159-3 | CN16×D1 | Gp1 |
| *Triticum aestivum* ssp. *aestivum* (AABBDD) | BAd160-6 | CN16×D1 | Gp1 |
| *Triticum aestivum* ssp. *aestivum* (AABBDD) | BAd161-5 | CN16×D1 | Gp1 |
| *Triticum aestivum* ssp. *aestivum* (AABBDD) | BAd162-5 | CN16×D1 | Gp3 |
| *Triticum aestivum* ssp. *aestivum* (AABBDD) | BAd163-2 | CN16×D1 | Gp1 |
| *Triticum aestivum* ssp. *aestivum* (AABBDD) | BAd164-5 | CN16×D1 | Gp1 |
| *Triticum aestivum* ssp. *aestivum* (AABBDD) | BFe164-6 | CN16×D1 | Gp1 |
| *Triticum aestivum* ssp. *aestivum* (AABBDD) | BAd167-6 | CN16×D1 | Gp1 |
| *Triticum aestivum* ssp. *aestivum* (AABBDD) | BAd168-3 | CN16×D1 | Gp1 |
| *Triticum aestivum* ssp. *aestivum* (AABBDD) | BAd169-4 | CN16×D1 | Gp1 |
| *Triticum aestivum* ssp. *aestivum* (AABBDD) | BAd170-1 | CN16×D1 | Gp1 |
| *Triticum aestivum* ssp. *aestivum* (AABBDD) | BAd170-2 | CN16×D1 | Gp1 |
| *Triticum aestivum* ssp. *aestivum* (AABBDD) | BAd170-4 | CN16×D1 | Gp1 |
| *Triticum aestivum* ssp. *aestivum* (AABBDD) | BAd170-6 | CN16×D1 | Gp1 |
| *Triticum aestivum* ssp. *aestivum* (AABBDD) | BAd173-5 | CN16×D1 | Gp1 |
| *Triticum aestivum* ssp. *aestivum* (AABBDD) | BAd174-1 | CN16×D1 | Gp1 |
| *Triticum aestivum* ssp. *aestivum* (AABBDD) | BAd174-6 | CN16×D1 | Gp1 |
| *Triticum aestivum* ssp. *aestivum* (AABBDD) | BAd175-3 | CN16×D1 | Gp1 |
| *Triticum aestivum* ssp. *aestivum* (AABBDD) | BAd175-6 | CN16×D1 | Gp1 |
| *Triticum aestivum* ssp. *aestivum* (AABBDD) | BAd175-7 | CN16×D1 | Gp1 |
| *Triticum aestivum* ssp. *aestivum* (AABBDD) | BAd176-4 | CN16×D1 | Gp1 |
| *Triticum aestivum* ssp. *aestivum* (AABBDD) | BAd177-2 | CN16×D1 | Gp1 |
| *Triticum aestivum* ssp. *aestivum* (AABBDD) | BAd177-6 | CN16×D1 | Gp1 |
| *Triticum aestivum* ssp. *aestivum* (AABBDD) | BAd178-2 | CN16×D1 | Gp1 |
| *Triticum aestivum* ssp. *aestivum* (AABBDD) | BAd178-6 | CN16×D1 | Gp1 |
| *Triticum aestivum* ssp. *aestivum* (AABBDD) | BAd179-4 | CN16×D1 | Gp1 |
| *Triticum aestivum* ssp. *aestivum* (AABBDD) | BAd180-3 | CN16×D1 | Gp1 |
| *Triticum aestivum* ssp. *aestivum* (AABBDD) | BAd180-5 | CN16×D1 | Gp1 |
| *Triticum aestivum* ssp. *aestivum* (AABBDD) | BAd181-1 | CN16×D1 | Gp1 |
| *Triticum aestivum* ssp. *aestivum* (AABBDD) | BAd182-3 | CN16×D1 | Gp1 |
| *Triticum aestivum* ssp. *aestivum* (AABBDD) | BAd182-5 | CN16×D1 | Gp1 |
| *Triticum aestivum* ssp. *aestivum* (AABBDD) | BAd183-3 | CN16×D1 | Gp1 |
| *Triticum aestivum* ssp. *aestivum* (AABBDD) | BAd183-4 | CN16×D1 | Gp1 |
| *Triticum aestivum* ssp. *aestivum* (AABBDD) | BAd184-1 | CN16×D1 | Gp2 |
| *Triticum aestivum* ssp. *aestivum* (AABBDD) | BAd185-1 | CN16×D1 | Gp1 |
| *Triticum aestivum* ssp. *aestivum* (AABBDD) | AdA116-(1) | BAd116-1×MM46 | Gp3 |
| *Triticum aestivum* ssp. *aestivum* (AABBDD) | AdA116-(3) | BAd116-1×MM46 | Gp3 |
| *Triticum aestivum* ssp. *aestivum* (AABBDD) | AdA116-(4) | BAd116-1×MM46 | Gp3 |
| *Triticum aestivum* ssp. *aestivum* (AABBDD) | AdA116-(5) | BAd116-1×MM46 | Gp3 |
| *Triticum aestivum* ssp. *aestivum* (AABBDD) | AdA116-(6) | BAd116-1×MM46 | Gp3 |
| *Triticum aestivum* ssp. *aestivum* (AABBDD) | AdA116-⑩ | BAd116-1×MM46 | Gp3 |
| *Triticum aestivum* ssp. *aestivum* (AABBDD) | AdA180-1 | BAd180-1×CM50 | Gp3 |
| *Triticum aestivum* ssp. *aestivum* (AABBDD) | AdA180-2 | BAd180-1×CM50 | Gp3 |
| *Triticum aestivum* ssp. *aestivum* (AABBDD) | AdA180-3 | BAd180-1×CM50 | Gp3 |
| *Triticum aestivum* ssp. *aestivum* (AABBDD) | AdA168-1 | BAd168-1×KCM2 | Gp3 |
| *Triticum aestivum* ssp. *aestivum* (AABBDD) | AdA168-2 | BAd168-1×KCM2 | Gp3 |
| *Triticum aestivum* ssp. *aestivum* (AABBDD) | AdA168-4 | BAd168-1×KCM2 | Gp3 |
| *Triticum aestivum* ssp. *aestivum* (AABBDD) | AdA168-5 | BAd168-1×KCM2 | Gp3 |
| *Triticum aestivum* ssp. *aestivum* (AABBDD) | AdA168-6 | BAd168-1×KCM2 | Gp3 |
| *Triticum aestivum* ssp. *aestivum* (AABBDD) | AdA168-9 | BAd168-1×KCM2 | Gp3 |
| *Triticum aestivum* ssp. *aestivum* (AABBDD) | AdA168-(1) | BAd168-1×KCM2 | Gp3 |
| *Triticum aestivum* ssp. *aestivum* (AABBDD) | AdA168-(10) | BAd168-1×KCM2 | Gp3 |
| *Triticum aestivum* ssp. *aestivum* (AABBDD) | AdA168-(11) | BAd168-1×KCM2 | Gp3 |
| *Triticum aestivum* ssp. *aestivum* (AABBDD) | AdA168-(12) | BAd168-1×KCM2 | Gp3 |
| *Triticum aestivum* ssp. *aestivum* (AABBDD) | AdA168-(14) | BAd168-1×KCM2 | Gp3 |
| *Triticum aestivum* ssp. *aestivum* (AABBDD) | AdA168-(15) | BAd168-1×KCM2 | Gp3 |
| *Triticum aestivum* ssp. *aestivum* (AABBDD) | AdA168-(16) | BAd168-1×KCM2 | Gp3 |
| *Triticum aestivum* ssp. *aestivum* (AABBDD) | AdA168-(17) | BAd168-1×KCM2 | Gp3 |
| *Triticum aestivum* ssp. *aestivum* (AABBDD) | AdA168-(18) | BAd168-1×KCM2 | Gp3 |
| *Triticum aestivum* ssp. *aestivum* (AABBDD) | AdA168-(19) | BAd168-1×KCM2 | Gp3 |
| *Triticum aestivum* ssp. *aestivum* (AABBDD) | AdA168-(2) | BAd168-1×KCM2 | Gp3 |
| *Triticum aestivum* ssp. *aestivum* (AABBDD) | AdA168-(20) | BAd168-1×KCM2 | Gp3 |
| *Triticum aestivum* ssp. *aestivum* (AABBDD) | AdA168-(21) | BAd168-1×KCM2 | Gp3 |
| *Triticum aestivum* ssp. *aestivum* (AABBDD) | AdA168-(3) | BAd168-1×KCM2 | Gp3 |
| *Triticum aestivum* ssp. *aestivum* (AABBDD) | AdA168-(4) | BAd168-1×KCM2 | Gp3 |
| *Triticum aestivum* ssp. *aestivum* (AABBDD) | AdA168-(5) | BAd168-1×KCM2 | Gp3 |
| *Triticum aestivum* ssp. *aestivum* (AABBDD) | AdA168-(6) | BAd168-1×KCM2 | Gp3 |
| *Triticum aestivum* ssp. *aestivum* (AABBDD) | AdA168-(7) | BAd168-1×KCM2 | Gp3 |
| *Triticum aestivum* ssp. *aestivum* (AABBDD) | AdA168-(8) | BAd168-1×KCM2 | Gp3 |
| *Triticum aestivum* ssp. *aestivum* (AABBDD) | AdA95-1 | BAd95-1×MM46 | Gp3 |
| *Triticum aestivum* ssp. *aestivum* (AABBDD) | AdA95-2 | BAd95-1×MM46 | Gp3 |
| *Triticum aestivum* ssp. *aestivum* (AABBDD) | AdA95-3 | BAd95-1×MM46 | Gp3 |
| *Triticum aestivum* ssp. *aestivum* (AABBDD) | AdA95-4 | BAd95-1×MM46 | Gp2 |
| *Triticum aestivum* ssp. *aestivum* (AABBDD) | AdA95-5 | BAd95-1×MM46 | Gp3 |
| *Triticum aestivum* ssp. *aestivum* (AABBDD) | AdA79-1 | BAd79-1×MM46 | Gp3 |
| *Triticum aestivum* ssp. *aestivum* (AABBDD) | AdA79-2 | BAd79-1×MM46 | Gp3 |
| *Triticum aestivum* ssp. *aestivum* (AABBDD) | AdA79-3 | BAd79-1×MM46 | Gp3 |
| *Triticum aestivum* ssp. *aestivum* (AABBDD) | AdA79-4 | BAd79-1×MM46 | Gp3 |
| *Triticum aestivum* ssp. *aestivum* (AABBDD) | AdA79-5 | BAd79-1×MM46 | Gp3 |
| *Triticum aestivum* ssp. *aestivum* (AABBDD) | AdA73-1 | BAd73-7×(YB58863×CY18) | Gp2 |
| *Triticum aestivum* ssp. *aestivum* (AABBDD) | AdA73-10 | BAd73-7×(YB58863×CY18) | Gp2 |
| *Triticum aestivum* ssp. *aestivum* (AABBDD) | AdA73-11 | BAd73-7×(YB58863×CY18) | Gp2 |
| *Triticum aestivum* ssp. *aestivum* (AABBDD) | AdA73-2 | BAd73-7×(YB58863×CY18) | Gp2 |
| *Triticum aestivum* ssp. *aestivum* (AABBDD) | AdA73-3 | BAd73-7×(YB58863×CY18) | Gp2 |
| *Triticum aestivum* ssp. *aestivum* (AABBDD) | AdA73-6 | BAd73-7×(YB58863×CY18) | Gp2 |
| *Triticum aestivum* ssp. *aestivum* (AABBDD) | AdA73-8 | BAd73-7×(YB58863×CY18) | Gp2 |
| *Triticum aestivum* ssp. *aestivum* (AABBDD) | AdA73-9 | BAd73-7×(YB58863×CY18) | Gp2 |
| *Triticum aestivum* ssp. *aestivum* (AABBDD) | AdA73-(2) | BAd73-7×(YB58863×CY18) | Gp2 |
| *Triticum aestivum* ssp. *aestivum* (AABBDD) | AdA73-(3) | BAd73-7×(YB58863×CY18) | Gp2 |
| *Triticum aestivum* ssp. *aestivum* (AABBDD) | AdA73-(4) | BAd73-7×(YB58863×CY18) | Gp2 |

^a^ Grouping by genotypic data using STRUCTURE.

**Supplementary Table2.**The distribution of DArT markers on the A, B, D, and whole genomes.

| Chr. | No. markers | | | | Map length (cM) | | | | Marker density (cM/marker) | | | |
| --- | --- | --- | --- | --- | --- | --- | --- | --- | --- | --- | --- | --- |
|  | A | B | D | Total | A | B | D | Total | A | B | D | Total |
| 1 | 693 | 1,708 | 240 | 2641 | 490.67 | 556.39 | 255.32 | 1302.38 | 0.71 | 0.33 | 1.06 | 0.49 |
| 2 | 794 | 1,350 | 544 | 2688 | 261.52 | 209.18 | 310.24 | 780.94 | 0.33 | 0.15 | 0.57 | 0.29 |
| 3 | 665 | 946 | 328 | 1939 | 275.51 | 306.21 | 291.21 | 872.93 | 0.41 | 0.32 | 0.89 | 0.45 |
| 4 | 780 | 345 | 52 | 1177 | 253.411 | 160.41 | 180.58 | 594.40 | 0.32 | 0.46 | 3.47 | 0.51 |
| 5 | 345 | 1,000 | 145 | 1490 | 297.81 | 305.64 | 250.09 | 853.53 | 0.86 | 0.31 | 1.72 | 0.57 |
| 6 | 619 | 592 | 202 | 1413 | 202.08 | 173.00 | 211.61 | 586.70 | 0.33 | 0.29 | 1.05 | 0.42 |
| 7 | 814 | 777 | 177 | 1768 | 306.71 | 273.93 | 369.62 | 950.27 | 0.38 | 0.35 | 2.09 | 0.54 |
| All | 4710 | 6718 | 1688 | 13116 | 2087.72 | 1984.75 | 1868.68 | 5941.15 | 0.44 | 0.30 | 1.11 | 0.45 |

Note: A total of 13116 mapped DArT markers with minor allele frequency ≥5% and missing ≤10%.

Chr, Chromosome.

cM, centimorgan.

**Supplementary Table3**.Percentage of pairwise markers in linkage disequilibrium (LD) across the A, B, D subgenomes, and the whole genome.

| Chromosome | Pairwise markers % (*p* < 0.001) | | | | Pairwise markers % (*p* < 0.001 and *r^2^* > 0.2) | | | |
| --- | --- | --- | --- | --- | --- | --- | --- | --- |
|  | A | B | D | Total | A | B | D | Total |
| 1 | 51.70 | 75.64 | 50.43 | 67.30 | 30.75 | 62.14 | 28.94 | 51.20 |
| 2 | 49.32 | 60.83 | 39.72 | 53.16 | 39.64 | 40.69 | 28.69 | 37.95 |
| 3 | 49.90 | 55.58 | 41.54 | 51.25 | 29.10 | 32.93 | 28.83 | 30.92 |
| 4 | 61.97 | 46.39 | 17.58 | 55.42 | 38.11 | 25.92 | 7.35 | 33.16 |
| 5 | 40.50 | 66.84 | 35.54 | 57.69 | 21.77 | 45.86 | 24.03 | 38.15 |
| 6 | 50.59 | 51.36 | 42.81 | 49.82 | 32.23 | 31.53 | 29.22 | 31.51 |
| 7 | 47.94 | 56.03 | 29.66 | 49.67 | 26.66 | 38.15 | 12.14 | 30.26 |
| All | 51.12 | 62.58 | 39.71 | 55.55 | 32.06 | 43.90 | 25.98 | 37.37 |

**Supplementary Table4.**Significant MTAs related to GPC detected by GWAS in this study based on BLUP values and GLM.

| QTL | Marker | Chromosomea | Position (cM)a | General linear model (GLM) | |
| --- | --- | --- | --- | --- | --- |
|  |  |  |  | -log10(*p*) | PVE (%) |
| *QGpc.cd1-1D.1* | 1105297 | 1D | 43.61 | 7.45 | 19.06 |
|  | 3944774 | 1D | 53.64 | 4.37 | 10.95 |
| *QGpc.cd1-1D.2* | 1094529 | 1D | 78.85 | 4.58 | 11.27 |
|  | 1120540 | 1D | 84.88 | 6.40 | 14.95 |
| *QGpc.cd1-1D.3* | 1116378 | 1D | 92.94 | 5.03 | 12.71 |
|  | 979304 | 1D | 101.51 | 5.32 | 13.48 |
| *QGpc.cd1-1D.4* | 3934740 | 1D | 140.88 | 5.16 | 12.55 |
| *QGpc.cd1-2A.1* | 1207352 | 2A | 125.28 | 4.47 | 11.22 |
|  | 1242803 | 2A | 127.65 | 4.59 | 10.58 |
|  | 1122854 | 2A | 129.30 | 4.59 | 10.58 |
|  | 1112129 | 2A | 130.95 | 4.42 | 11.00 |
|  | 1862406 | 2A | 130.95 | 4.47 | 11.29 |
|  | 3025579 | 2A | 130.95 | 4.45 | 11.31 |
|  | 1096041 | 2A | 131.57 | 4.42 | 10.93 |
|  | 1137907 | 2A | 132.96 | 4.37 | 11.01 |
| *QGpc.cd1-2A.2* | 1076002 | 2A | 137.60 | 5.63 | 13.93 |
|  | 1203032 | 2A | 137.60 | 4.41 | 11.05 |
|  | 1725676 | 2A | 137.60 | 4.55 | 10.54 |
|  | 3950937 | 2A | 137.60 | 4.12 | 10.41 |
|  | 1105928 | 2A | 138.43 | 7.07 | 18.00 |
|  | 3025920 | 2A | 139.37 | 4.53 | 11.22 |
|  | 1027267 | 2A | 139.90 | 7.16 | 18.32 |
| *QGpc.cd1-2A.3* | 1228295 | 2A | 234.11 | 4.59 | 11.09 |
| *QGpc.cd1-2B.1* | 1116535 | 2B | 3.93 | 7.84 | 20.19 |
|  | 1267019 | 2B | 4.39 | 6.46 | 15.19 |
|  | 1092629 | 2B | 7.26 | 5.55 | 13.99 |
|  | 1235214 | 2B | 7.26 | 7.74 | 18.52 |
|  | 1088696 | 2B | 8.70 | 7.89 | 19.93 |
|  | 2311069 | 2B | 8.70 | 7.57 | 19.50 |
|  | 3022213 | 2B | 8.70 | 8.42 | 21.35 |
| *QGpc.cd1-2B.2* | 1020261 | 2B | 69.20 | 4.23 | 10.05 |
| *QGpc.cd1-2D.1* | 1102577 | 2D | 228.90 | 4.13 | 10.30 |
| *QGpc.cd1-2D.2* | 1114546 | 2D | 261.71 | 4.36 | 11.06 |
|  | 1116168 | 2D | 264.00 | 4.17 | 10.19 |
| *QGpc.cd1-3A.1* | 1217997 | 3A | 77.16 | 5.42 | 13.56 |
| *QGpc.cd1-3A.2* | 1154276 | 3A | 256.32 | 4.34 | 10.06 |
| *QGpc.cd1-3B.1* | 996936 | 3B | 94.94 | 5.26 | 12.99 |
|  | 1027428 | 3B | 94.94 | 4.43 | 10.81 |
|  | 1034676 | 3B | 94.94 | 4.52 | 10.46 |
|  | 1169595 | 3B | 94.94 | 4.88 | 12.30 |
|  | 1695648 | 3B | 94.94 | 7.66 | 18.03 |
|  | 4909733 | 3B | 94.94 | 5.43 | 12.87 |
|  | 1083125 | 3B | 94.98 | 5.72 | 13.34 |
|  | 1125082 | 3B | 95.26 | 4.67 | 10.84 |
|  | 1008114 | 3B | 95.66 | 6.07 | 15.63 |
|  | 2255756 | 3B | 97.24 | 5.17 | 12.20 |
|  | 2299699 | 3B | 97.24 | 5.90 | 14.44 |
|  | 5971202 | 3B | 97.24 | 4.48 | 10.74 |
|  | 1036848 | 3B | 97.41 | 5.32 | 13.30 |
|  | 1079842 | 3B | 98.97 | 5.91 | 15.02 |
|  | 1125477 | 3B | 98.97 | 4.38 | 10.97 |
| *QGpc.cd1-3B.2* | 1230212 | 3B | 105.52 | 6.02 | 14.06 |
|  | 1237956 | 3B | 106.00 | 5.28 | 12.25 |
|  | 1289877 | 3B | 113.11 | 6.39 | 16.36 |
| *QGpc.cd1-3B.3* | 3021856 | 3B | 135.98 | 4.48 | 11.38 |
| *QGpc.cd1-3B.4* | 5325117 | 3B | 185.87 | 4.12 | 10.08 |
|  | 3033906 | 3B | 186.28 | 4.22 | 9.90 |
| *QGpc.cd1-3B.5* | 1217090 | 3B | 198.30 | 4.64 | 11.52 |
|  | 2277868 | 3B | 198.30 | 4.42 | 10.28 |
| *QGpc.cd1-3B.6* | 3031867 | 3B | 218.99 | 5.90 | 14.45 |
| *QGpc.cd1-3B.7* | 1101435 | 3B | 251.88 | 4.17 | 10.41 |
|  | 1379552 | 3B | 260.22 | 5.87 | 13.87 |
| *QGpc.cd1-3D.1* | 2242056 | 3D | 30.91 | 6.86 | 17.47 |
| *QGpc.cd1-3D.2* | 1061456 | 3D | 88.72 | 4.77 | 11.94 |
| *QGpc.cd1-3D.3* | 1123163 | 3D | 113.35 | 5.79 | 14.41 |
| *QGpc.cd1-3D.4* | 1139378 | 3D | 155.88 | 6.20 | 15.98 |
| *QGpc.cd1-3D.5* | 2251393 | 3D | 285.53 | 6.30 | 15.53 |
| *QGpc.cd1-4B.1* | 1313382 | 4B | 13.83 | 6.04 | 14.69 |
| *QGpc.cd1-4B.2* | 1106324 | 4B | 60.12 | 4.65 | 11.55 |
| *QGpc.cd1-4D* | 2243585 | 4D | 72.55 | 5.35 | 13.05 |
| *QGpc.cd1-5A* | 1211121 | 5A | 97.00 | 4.23 | 10.37 |
| *QGpc.cd1-5B.1* | 1104807 | 5B | 16.29 | 6.71 | 17.29 |
|  | 979512 | 5B | 22.17 | 4.37 | 10.03 |
| *QGpc.cd1-5B.2* | 1213013 | 5B | 27.09 | 4.82 | 11.98 |
|  | 989907 | 5B | 29.60 | 7.80 | 18.56 |
|  | 990408 | 5B | 30.50 | 7.52 | 18.64 |
|  | 2290450 | 5B | 30.50 | 6.93 | 16.90 |
|  | 2373184 | 5B | 32.89 | 7.06 | 16.92 |
|  | 2254812 | 5B | 35.47 | 6.93 | 17.54 |
|  | 2293506 | 5B | 37.56 | 7.62 | 18.78 |
| *QGpc.cd1-5B.3* | 1087699 | 5B | 47.44 | 7.25 | 18.23 |
|  | 1123759 | 5B | 49.46 | 8.11 | 19.87 |
|  | 1135294 | 5B | 49.46 | 4.17 | 10.19 |
|  | 1240690 | 5B | 49.46 | 4.77 | 11.78 |
|  | 2374408 | 5B | 49.46 | 6.17 | 15.49 |
|  | 2295246 | 5B | 50.14 | 9.45 | 22.48 |
|  | 1097189 | 5B | 50.90 | 5.01 | 12.33 |
|  | 3025777 | 5B | 50.90 | 4.71 | 11.49 |
|  | 3533063 | 5B | 50.90 | 4.17 | 9.93 |
|  | 1000420 | 5B | 52.21 | 6.03 | 15.33 |
|  | 1129545 | 5B | 52.21 | 4.44 | 10.85 |
|  | 1261730 | 5B | 52.21 | 6.25 | 16.01 |
|  | 1064158 | 5B | 55.28 | 8.35 | 20.43 |
|  | 1131454 | 5B | 55.28 | 6.86 | 17.57 |
|  | 1302570 | 5B | 55.28 | 8.93 | 21.89 |
|  | 9724914 | 5B | 55.55 | 6.94 | 16.34 |
|  | 1107715 | 5B | 55.97 | 7.53 | 18.99 |
|  | 1110973 | 5B | 55.97 | 7.17 | 17.59 |
|  | 3023877 | 5B | 56.97 | 6.49 | 16.72 |
| *QGpc.cd1-5B.4* | 1070542 | 5B | 58.65 | 8.42 | 21.36 |
|  | 1862481 | 5B | 58.65 | 4.41 | 10.19 |
| *QGpc.cd1-5B.5* | 5331639 | 5B | 209.60 | 5.83 | 14.73 |
| *QGpc.cd1-5D* | 1269698 | 5D | 154.90 | 5.23 | 12.97 |
| *QGpc.cd1-6A.1* | 1258213 | 6A | 1.24 | 6.54 | 15.29 |
| *QGpc.cd1-6A.2* | 1207135 | 6A | 54.76 | 4.27 | 10.08 |
|  | 1114701 | 6A | 57.25 | 4.62 | 10.72 |
| *QGpc.cd1-6B.1* | 3385102 | 6B | 12.86 | 5.06 | 11.81 |
|  | 1228001 | 6B | 19.85 | 7.01 | 17.10 |
|  | 979061 | 6B | 21.22 | 7.38 | 18.55 |
| *QGpc.cd1-6B.2* | 1166413 | 6B | 25.24 | 5.77 | 14.65 |
|  | 1054380 | 6B | 25.62 | 6.42 | 15.65 |
|  | 1099168 | 6B | 25.62 | 6.85 | 16.72 |
|  | 2267344 | 6B | 25.62 | 6.92 | 16.97 |
|  | 1120297 | 6B | 27.67 | 5.86 | 14.71 |
|  | 2249897 | 6B | 27.67 | 4.66 | 11.19 |
|  | 1208994 | 6B | 32.52 | 5.45 | 13.82 |
|  | 2275505 | 6B | 34.50 | 4.46 | 11.11 |
| *QGpc.cd1-6B.3* | 1118076 | 6B | 45.17 | 6.78 | 17.47 |
| *QGpc.cd1-6D.1* | 1070747 | 6D | 0.77 | 4.78 | 12.05 |
| *QGpc.cd1-6D.2* | 1235995 | 6D | 114.20 | 5.19 | 12.80 |
| *QGpc.cd1-7B.1* | 2291781 | 7B | 194.88 | 5.66 | 13.27 |
|  | 3034442 | 7B | 201.67 | 6.80 | 17.42 |
|  | 1098577 | 7B | 202.18 | 4.44 | 10.77 |
|  | 1228239 | 7B | 202.18 | 6.55 | 16.27 |
|  | 1091674 | 7B | 202.20 | 6.79 | 15.98 |
|  | 1102261 | 7B | 202.20 | 6.76 | 15.99 |
|  | 1104863 | 7B | 202.20 | 6.54 | 15.48 |
|  | 1128536 | 7B | 202.20 | 5.91 | 14.56 |
|  | 1130305 | 7B | 203.43 | 5.47 | 13.04 |
|  | 997593 | 7B | 203.81 | 7.15 | 17.23 |
|  | 1109823 | 7B | 204.25 | 6.18 | 15.14 |
| *QGpc.cd1-7B.2* | 3027463 | 7B | 208.14 | 7.35 | 18.25 |
|  | 1166404 | 7B | 216.46 | 9.07 | 22.79 |
|  | 1087709 | 7B | 217.05 | 4.65 | 11.33 |
|  | 1266721 | 7B | 217.05 | 4.30 | 10.40 |
|  | 1083673 | 7B | 217.18 | 8.47 | 19.78 |
| *QGpc.cd1-7B.3* | 1127602 | 7B | 222.83 | 8.08 | 19.43 |
|  | 1087698 | 7B | 230.66 | 4.30 | 10.21 |
| *QGpc.cd1-7B.4* | 1096464 | 7B | 250.64 | 4.14 | 9.93 |
| *QGpc.cd1-7D.1* | 1093788 | 7D | 110.59 | 4.62 | 11.54 |
| *QGpc.cd1-7D.2* | 1088336 | 7D | 124.19 | 4.37 | 10.93 |

^a^ Chromosomal location information based on the wheat consensus map version 3.0 (http://www.diversityarrays.com/sequence-maps).

PVE, phenotypic variation explained.

**Supplementary Table5.** List of predicated candidate genes for GPC.

| QTL | Maker | | Chromosome | | IWGSC v1.0 database | | Wild emmer database | |
| --- | --- | --- | --- | --- | --- | --- | --- | --- |
|  | GLM | MLM | Consistent map | IWGSC | Gene | Gene Description | Gene | Gene Description |
| *QGpc.cd1-1D.1* | 1105297 | \ | 1D | 1DS | TraesCS1D01G029200.2 | Disease resistance protein (NBS-LRR class) family | \ | \ |
| *QGpc.cd1-2A.2* | 1203032 | \ | 2A | 2AL | TraesCS2A01G328100.1 | NAC domain-containing protein (No apical meristem (NAM) protein) | TRIDC2AG048870.1 | Pentatricopeptide repeat-containing protein |
|  | 1725676 | \ | 2A | 2AL | TraesCS2A01G322500.1 | Cytochrome P450 | TRIDC2AG049560.1 | Cytochrome P450 superfamily protein |
|  | 3950937 | \ | 2A | 2AL | TraesCS2A01G324500.1 | Methyltransferase | TRIDC2AG049350.1 | Methyltransferase type 11 |
| *QGpc.cd1-2B.1* | 1116535 | 1116535 | 2B | 2BS | TraesCS2B01G002700 | ABA-responsive binding factor | TRIDC2BG001520.1 | Ethylene-responsive transcription factor 12 |
|  |  |  |  |  | TraesCS2B01G003000 | Cytochrome P450 family protein | TRIDC2BG001480.1 | Cytochrome P450 superfamily protein |
|  |  |  |  |  | TraesCS2B01G003300LC.1 | acetyl-CoA carboxylase 1 | TRIDC2BG001550.1 | Cytochrome c oxidase subunit 1 |
|  | 1088696 | 1088696 | 2B | 2BS | TraesCS2B01G002300LC | Leucine-rich repeat receptor-like protein kinase family protein | TRIDC2BG001580.1 | Cytochrome c oxidase subunit 2 |
|  |  |  |  |  | TraesCS2B01G003000LC | Maturase K | \ | \ |
|  | 3022213 | 3022213 | 2B | 2BS | TraesCS2B01G003600LC.1 | ABA-responsive binding factor | \ | \ |
|  |  |  |  |  | TraesCS2B01G004500LC.1 | S-locus lectin protein kinase family protein | \ | \ |
| *QGpc.cd1-3B.1* | 4909733 | \ | 3B | \ | TraesCS3B01G151500.1 | MYB transcription factor | TRIDC3BG021420.2 | Myb transcription factor |
| *QGpc.cd1-3B.7* | 1379552 | \ | 3B | \ | TraesCS3B01G576100.1 | HIT-type zinc finger family protein | TRIDC3BG083210.1 | HIT-type Zinc finger family protein |
| *QGpc.cd1-3D.3* | 1123163 | \ | 3D | 3DL | TraesCS3D01G255800.1 | Peptidase S8 | \ | \ |
| *QGpc.cd1-5A* | 1211121 | \ | 5A | 5AL | TraesCS5A01G270700.1 | Cytochrome P450 | TRIDC5AG040970.2 | Cytochrome P450 superfamily protein |
| *QGpc.cd1-5B.1* | 1104807 | \ | 5B | 5BS | TraesCS5B01G015400.1 | Protein phosphatase 2C family protein | \ | \ |
|  | 979512 | \ | 5B | 5BS | TraesCS5B01G027900LC.1 | Leucine-rich repeat receptor-like protein kinase family protein | \ | \ |
| *QGpc.cd1-5B.2* | 989907 | \ | 5B | 5BL | TraesCS5B01G486400.1 | Ubiquitin family protein | TRIDC5BG072940.1 | Ubiquitin-like superfamily protein |
|  | 990408 | \ | 5B | 5BS | \ | \ | TRIDC5BG040050.1 | alpha/beta-Hydrolases superfamily protein |
|  | 2293506 | \ | 5B | 5BS | TraesCS5B01G047400LC.1 | proline transporter 1 | \ | \ |
| *QGpc.cd1-5B.3* | 1261730 | \ | 5B | 5BS | \ | \ | TRIDC5BG008360.3 | copper ion binding |
|  | 2295246 | \ | 5B | \ | TraesCS5B01G077300LC.1 | glutathione S-transferase THETA 3 | \ | \ |
|  | 1302570 | \ | 5B | 5BL | TraesCS5B01G276400LC.1 | Beta-galactosidase | TRIDC5BG028320.1 | Leucine-rich repeat receptor-like protein kinase family protein |
|  | 1064158 | \ | 5B | 5BL | TraesCS5B01G278900LC.1 | NAC domain protein (NAM protein) | \ | \ |
|  | 1110973 | \ | 5B | 5BL | TraesCS5B01G167900.1 | Cysteine proteinase | TRIDC5BG029410.1 | Para-aminobenzoate synthase |
| *QGpc.cd1-6A.1* | 1258213 | \ | 6A | 6AS | TraesCS6A01G004600LC.1 | Nitrogenase-stabilizing/protective protein NifW | TRIDC6AG000490.1 | NAC domain containing protein 53 |
| *QGpc.cd1-6B.1* | 1228001 | \ | 6B | 6BS | TraesCS6B01G049300LC.1 | rRNA N-glycosidase | \ | \ |
| *QGpc.cd1-6B.2* | 1166413 | \ | 6B | 6BS | TraesCS6B01G045700.1 | High affinity nitrate transporter | TRIDC6BG005700.1 | High affinity nitrate transporter 2.6 |
|  | 1208994 | \ | 6B | 6BS | TraesCS6B01G063300.1 | GMP synthase | TRIDC6BG007960.1 | GMP synthase |
| *QGpc.cd1-6B.3* | 1118076 | \ | 6B | 6BS | TraesCS6B01G128000.2 | Zinc transporter | TRIDC6BG018040.1 | Zinc transporter 2 |
|  |  |  |  |  | TraesCS6B01G128100.1 | Kinase-like protein | TRIDC6BG018050.1 | protein kinase family protein |
| *QGpc.cd1-7B.1* | 1104863 | \ | 7B | 7BL | TraesCS7B01G440000.2 | Protein tyrosine kinase | TRIDC7BG069500.3 | Protein kinase superfamily protein |
|  | 1128536 | \ | 7B | 7BL | TraesCS7B01G439300.1 | Cytochrome P450 family protein | TRIDC7BG067940.1 | Cytochrome P450 superfamily protein |
| *QGpc.cd1-7B.2* | 3027463 | \ | 7B | 7BL | TraesCS7B01G449500.1 | Protein tyrosine kinase | \ | \ |
|  | 1166404 | 1166404 | 7B | 7BL | TraesCS7B01G450600 | Processing peptidase | TRIDC7BG060020.1 | Eukaryotic aspartyl protease family protein |
|  |  |  |  |  | TraesCS7B01G450100 | Senescence/dehydration-associated-like protein | \ | \ |
|  |  |  |  |  | TraesCS7B01G757900LC | Endonuclease/exonuclease/phosphatase family protein | \ | \ |
|  | 1087709 | \ | 7B | 7BL | \ | \ | TRIDC7BG071930.1 | Heavy metal transport |
| *QGpc.cd1-7D.1* | 1093788 | \ | 7D | 7DS | TraesCS7D01G135800LC.1 | AMP deaminase 3 | \ | \ |
| *QGpc.cd1-7D.2* | 1088336 | \ | 7D | 7DS | TraesCS7D01G162600LC.1 | Respiratory nitrate reductase 2 gamma chain | \ | \ |

Note: Chromosomal location information based on the wheat consensus map version 3.0 (http://www.diversityarrays.com/sequence-maps) and the best hit on IWGSC (the International Wheat Genome Sequencing Consortium), respectively. "\", data unavailable.
